# Effect of vaginal microbiota on pregnancy outcomes of women from Northern China who conceived after IVF

**DOI:** 10.3389/fendo.2023.1200002

**Published:** 2023-07-18

**Authors:** Yu Tong, Qiang Sun, Xiaoguang Shao, Zhijian Wang

**Affiliations:** ^1^ Department of Obstetrics and Gynecology, Nanfang Hospital, Southern Medical University, Guangzhou, Guangdong, China; ^2^ Department of Obstetrics and Gynecology, Dalian Municipal Women and Children’s Medical Group, Dalian, Liaoning, China

**Keywords:** pregnancy, vaginal microbiome, preterm birth, *in vitro* fertilization (IVF), pregnancy outcomes

## Abstract

**Objective:**

This study aimed to investigate the correlation between vaginal microbiota and pregnancy outcomes of women who achieved pregnancy *via in vitro* fertilization (IVF) in Northern China, and to determine a biomarker for evaluation of the risk of preterm births in these women.

**Methods:**

In total, 19 women from Northern China women who conceived after IVF and 6 women who conceived naturally were recruited in this study. The vaginal samples of the healthy participants were collected throughout pregnancy, that is, during the first, second, and third trimesters. The V3–V4 region of 16S rRNA was used to analyze the vaginal microbiome, and the bioinformatic analysis was performed using QIIME Alpha and Beta diversity analysis.

**Results:**

Either IVF group or Natural conception group, bacterial community diversities and total species number of vagnal samples from who delivered at term were significantly higher than those who delivered before term. Low abundance of vaginal bacteria indicates an increased risk of preterm delivery. Further, more abundant vaginal bacteria was found in first trimesters instead of the next two trimesters. Vignal samples collected during first trimester showed richer differences and more predictive value for pregnancy outcoes. In addition, the diversity of the vaginal bacterial community decreased as the gestational age increased, in all samples. *Alloscardovia* was only found in participants who conceived after IVF, and the percentage of *Alloscardovia* in viginal samples of normal delivery group is much higher than the samples from preterm delivery group.*Vobrio* specifically colonized in vagina of pregnant woman in AFT group (those who conceived after IVF (A), first trimester (F), and delivered at term (T)) and *Sporosarcina* was detected only in women with AFT and AST (those who conceived after IVF (A), second trimester (S), and delivered at term (T)). These data indicates that *Alloscardovia, Vobrio* and *Sporosarcina* have great potential in predicting pregnancy outcomes who pregnanted by vitro fertilization

**Conclusions:**

Vaginal microbiota were more stable in women who conceived naturally and those who carried pregnancy to term. *Oceanobacillus* might act as a positive biomarker, whereas *Sulfurospirillum* and *Propionispira* may act as negative biomarkers for the risk of preterm birth.

## 1 Introduction

Preterm birth, defined as delivery of the infant before 37 weeks of gestation, is a major contributor to infant death and neonatal mortality, which is a global public health concern (1–3). Human reproduction is an inefficient process, and couples opt for conception through *in vitro* fertilization (IVF) for several reasons such as higher maternal age and failure to conceive naturally. However, IVF (4, 5) is associated with increased rates of multiple gestations and an increased incidence of preterm birth (6, 7).

The World Health Organization has reported that approximately 15 million babies are “born too early” (8–10). The risk of preterm birth is multifactorial, and reproductive tract infection is considered a major triggering factor (11). The costs associated with preterm births have been estimated to rise to $26 billion since the year 2005 (12); thus, it is important to determine an efficient approach to detect the risk of preterm birth during gestation.

Vaginal microbiomes have been proposed to play some roles in risk evaluation and disease diagnosis in women (13). Van der Wijgert et al. reviewed studies reported on molecular vaginal microbiota (VMB) for 6 year, and found that molecular techniques such as sequencing, PCR(polymerase chain reaction), DNA fingerprinting, and DNA hybridization are effective to characterize the VMB, and that lactobacilli-dominated VMB are associated with a healthy vaginal microenvironment (14, 15). Bacterial vaginosis, described as a polybacterial dysbiosis, is a risk factor for preterm births (16–19). A previous study showed that abnormal vaginal microorganisms can negatively affect the pregnancy rate among women who conceive after IVF (18), and that VMB present on the day of embryo transfer significantly affect the pregnancy outcome of IVF (live birth/no live birth) (20), indicating that some VMB might act as positive or negative biomarkers for the risk of preterm births.

It is difficult to identify patients with a risk of preterm birth among those who undergo IVF (17). The microbial composition in the vagina of women who conceive following IVF is still unclear, and whether differences exist in the vaginal bacterial communities during the first, second, and third trimesters in women who conceive naturally and those who conceive *via* IVF is poorly understood. Vaginal bacteria vary among women from different races and ethnicities (21, 22). Previous studies in this regard have mostly focused on women from Europe and America. Few studies focusing on African populations have been reported (23, 24), mainly in Nigeria; limited studies have been conducted on women in China.

In this study, we investigated whether the composition of VMB among women in Northern China who conceived naturally differed from the composition among women who conceived after IVF, as well as whether the composition of VMB among women who subsequently delivered before term differed from that among women who delivered at term. Through this study, we aimed to unveil the changes in VMB in the first, second, and third trimesters of pregnancy to determine the most crucial period and the key biomarkers of vaginal bacteria.

## 2 Materials and methods

### 2.1 Sampling information

The study participants were women from Dalian, a northern coastal city in China. Participants were selected from women who underwent routine prenatal examination at the Dalian Women’s and Children’s Medical Center between 2017 and 2019. The mothers were enrolled at the hospital admission for delivery and provided written informed consent for participation. The study protocol was approved by the Dalian Women’s and Children’s Medical Center (approval number 20160021). All operations and evaluations were performed by the same doctor. In total, 19 women had conceived after IVF after a long protocol of conventional solutions of fresh-cycle IVF and conventional luteal support after 1 week; 6 of these delivered before term, whereas 13 delivered at term. Six pregnant women had visited the obstetrics and gynecology clinic for a routine pregnancy test during the same period; three of these women delivered before term, whereas three delivered at term.

The inclusion criteria for this study were as follows: (1) No symptoms of vaginitis, single live births, and primipara; (2) abstinence from sex for 2 weeks, no antibiotic use, no medication history related to the vulva and vagina for 1 month prior to the study, and no history of systemic use of hormone drugs and immunosuppressants; (3) no history of pregnancy complications such as vaginitis, gestational hypertension disease, gestational diabetes mellitus, or pregnancy with abnormal thyroid function; and (4) no history of smoking, drinking, or drug consumption.

Vaginal swab specimens were collected throughout the prenatal check-ups of the healthy participants in all three trimesters of pregnancy. The swabs were obtained at routine prenatal visits, and by rotating a sterilized swab five times along the vaginal lumen in a circular motion. Speculum was not used. No occurrences of premature rupture of membranes was observed, and no participant was administered intravenous antibiotics during delivery. The participants were divided into those who conceived after IVF and those who conceived naturally (controls), and according to the mode of delivery, as full term (≥37 weeks) and preterm (<37 weeks). The collected swabs were placed into sterile tubes by trained research staff and stored at −80°C for DNA extraction.

The vaginal swabs were collected at least in triplicate at three time points: first trimester (F, 10–13 + 6 weeks), second trimester (S, 20–27 + 6 weeks), and third trimester (T, 28–33 + 6 weeks) of pregnancy. The presence of specific vaginal bacterial communities in any single gestational period in participants who delivered before term (P, six participants in IVF pregnancy group, three participants in natural pregnancy group) was compared with that in participants who delivered at term (T, 13 participants in IVF pregnancy group, 3 participants in natural pregnancy group).

### 2.2 IVF protocol

The standard long IVF regimen was used. A gonadotropin-releasing hormone (GnRH) agonist regimen was the main protocol used in this study, and all patients underwent embryo transfer in fresh cycles. Controlled ovarian hyper-stimulation, oocyte retrieval, and embryo transfer were carried out. Participants were treated with downregulation from the mid-luteal phase of the previous cycle. When the pituitary reached desensitization, recombinant FSH was begun at 150–225 IU/day. Human chorionic gonadotropin (hCG) was given (4000–10,000 IU) once two or more follicles had reached a size of 18 mm. Oocytes were extracted 34–36 h after the hCG trigger, and this was followed by intracytoplasmic sperm injection (ICSI). Participants were injected progesterone (lot NO. 1220507 Shanghai General Pharma, China) at 40 mg/day 48 h after oocytes fertilization. The progesterone supplementation was continued until 10 weeks of gestation after pregnancy was achieved.

### 2.3 Genomic DNA extraction and 16S rDNA gene sequencing analysis

Genomic DNA was extracted using cetyltrimethylammonium bromide; the concentration and purity of DNA were detected by 1% agarose gel electrophoresis. The appropriate amount of sample was placed into the tube and diluted with sterile water to 1 ng/μL. High-throughput sequencing technology was used to sequence the V3–V4 region of the 16S rRNA gene, which was amplified used a specific primer with the barcode 314F-806R (V3V4 primers: 314F-5′ CCTAYGGGRBGCASCAG 806R5′ GGACTACNNGGGTATCTAAT) Phusion^®^ High-Fidelity PCR Master Mix (New England Biolabs) was used for PCR amplification according to the selection of the sequencing region.

### 2.4 PCR product purification

PCR products were mixed with the same volume of 1× loading buffer and detected on 2% agarose gel electrophoresis. Sample strips of 400–450 bp were chosen for further experiments. The PCR products were purified using Qiagen Gel Extraction Kit (Qiagen, Hilden, Germany).

### 2.5 Library preparation and sequencing

TruSeq^®^ DNA PCR-Free Sample Preparation Kit (Illumina, San Diego, CA, USA) was used to generate sequencing libraries, and their quality was analyzed using the Qubit@ 2.0 Fluorometer (Life Technologies, Thermo Fisher Scientific, Waltham, MA, USA) and Agilent Bioanalyzer 2100 system. The library was sequenced on Illumina HiSeq2500 platform, and finally, 250-bp paired-end reads were generated.

### 2.6 Sequencing data analyses

The reads were merged by FLASH to obtain raw tags (V1.2.7) (25), and quality filtering was performed to obtain high-quality clean tags (26) by QIIME (V1.7.0) (27). The tags were compared with the Gold database, and finally, the effective tags were obtained using UCHIME algorithm (28, 29). Uparse software (Uparse v7.0.1001) was used to analyze the sequences (30), which were assigned to the same operational taxonomic units (OTUs) once the similarities were more than 97%. The Silva Database (31) was used based on the Ribosomal Databases Project classifier (Version 2.2) algorithm to annotate taxonomic information (32). MUSCLE software (Version 3.8.31) was used to align multiple sequences, to further analyze the phylogenetic relationships of OTUs, and to determine the dominant species in the different groups (33). Based on further information about the abundance of normalized OTUs, we subsequently analyzed their alpha/beta diversity.

### 2.7 Alpha and beta diversity analysis

Alpha diversity was exhibited by six indices, namely Observed-species, Chao1, Shannon, Simpson, ACE, and Good’s Coverage, which were calculated with QIIME (Version 1.7.0) and displayed using R software (Version 2.15.3). Beta diversity was calculated by QIIME software (Version 1.7.0) and R software (Version 2.15.3). Species analysis with significant differences between groups was performed using Student’s *t*-test and mapping between groups was done using R software (Version 2.15.3).

### 2.8 Date availability statement

We Uploaded the raw 16S rRNA gene sequencing data to the National Center for Biotechnology Information (accession no. PRJNA728871).

## 3 Results

### 3.1 Basic participant and specimen characteristics

The details of the participants are stated in **Table 1**. The average age of participants who conceived *via* IVF(A)was 31.42 ± 10.23 years, and that of those who conceived naturally (B) was 31.50 ± 4.52 years (P > 0.05). The mean BMI of the participants in Group A was 26.57 ± 2.03 kg/m^2^ and that of participants in Group B was 27.48 ± 0.95 kg/m^2^ (P > 0.05); The mean fetal weight in Group A was 3157.89 ± 628.55 g and that in Group B was 2991.67 ± 452.12 g (P > 0.05).

**Table 1:** Basic information of participants.

	Group A (n=19)	Group B (n=6)	P-value
Age (y)	31.42 ± 10.23	31.50 ± 4.52	P>0.05
BMI	26.57 ± 2.03	27.48 ± 0.95	P>0.05
Gestation (wk)	37.84 ± 2.54	33.75 ± 2.61	P>0.05
Fetal weight (g)	2486.00 ± 628.55	2991.67 ± 452.12	P>0.05

Group A means who conceived via IVF; Group B who conceived naturally.

The details of the specimens collected from the participants who conceived *via* IVF (Group A, 19 participants) and those who conceived naturally (Group B, 6 participants) are stated in **Table 2**. The participants were further divided into women who delivered before term (P) and those who delivered at term (T).

**Table 2:** Participant groups along with their abbreviations*.

Pregnancy mode	First word	Second word			Delivery mode	Third word
		First trimester	Second trimester	Third trimester		
IVF	A	F	S	T	Term	T
Natural	B	Preterm	P			

Designation of the bigger group contains only the first and the third word.

**Figure 1:**
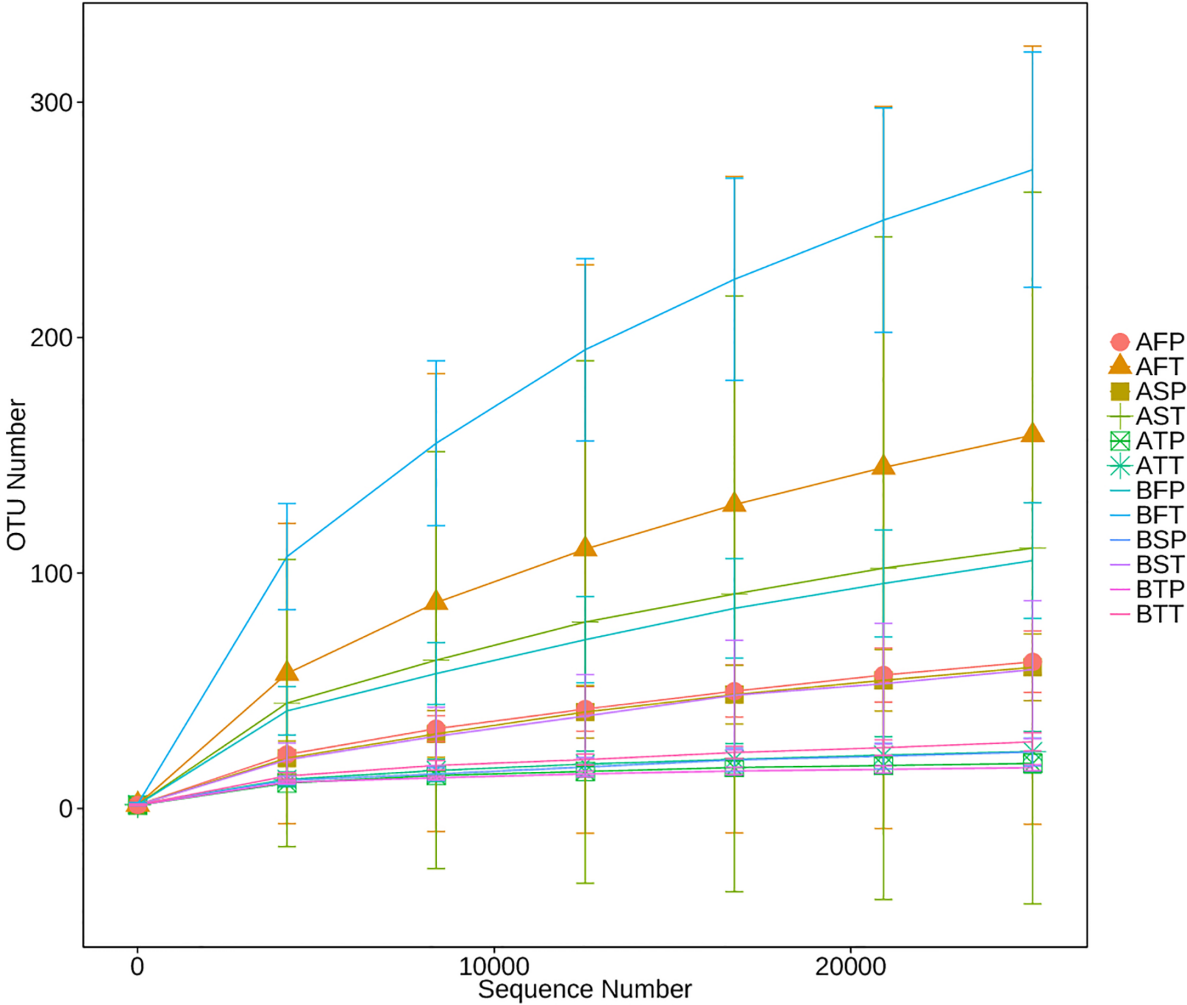
Rarefaction analysis of different samples. Rarefaction curves of operational taxonomic units across different samples, which indicated the saturation plateau of the different samples. Samples were designated by three letters. For the first letter, **(A)** indicates IVF pregnancy, **(B)** indicates natural pregnancy; for the second letter, F indicates early gestation, S indicates mid-gestation, T indicates late gestation; for the third letter, P indicates preterm delivery, T indicates term delivery.

16S rRNA of each sample was analyzed using high-throughput sequencing technology. The rarefaction curves tended to approach saturation in all samples except for sample BFT (women who conceived naturally (B), first trimester (F), and delivered at term (T))(**Figure 1**); Good’s Coverage revealed that 99%–100% of the species were detected in all samples, suggesting that the data were suitable for further analyses. The details are shown in **Table 3**.

**Table 3:** Basic information of different samples.

Group	Observed species	Shannon	Simpson	Chao1	ACE	Good’s coverage
AFP	62	0.546	0.181	117.174	142.635	0.999
AFT	158	0.617	0.135	270.588	302.016	0.997
ASP	60	0.468	0.121	99.868	111.116	0.999
AST	110	0.555	0.122	182.460	199.882	0.998
ATP	19	0.468	0.153	21.338	24.101	1.000
ATT	24	0.589	0.205	33.044	35.261	1.000
BFP	105	0.575	0.119	186.236	240.106	0.998
BFT	271	0.993	0.177	447.082	479.467	0.995
BSP	24	0.424	0.117	36.133	40.399	1.000
BST	59	0.426	0.111	95.988	114.137	0.999
BTP	17	0.449	0.123	20.500	22.917	1.000
BTT	28	0.550	0.182	41.028	47.717	1.000

### 3.2 Bacterial characteristics changed throughout pregnancy

**Figure 2:**
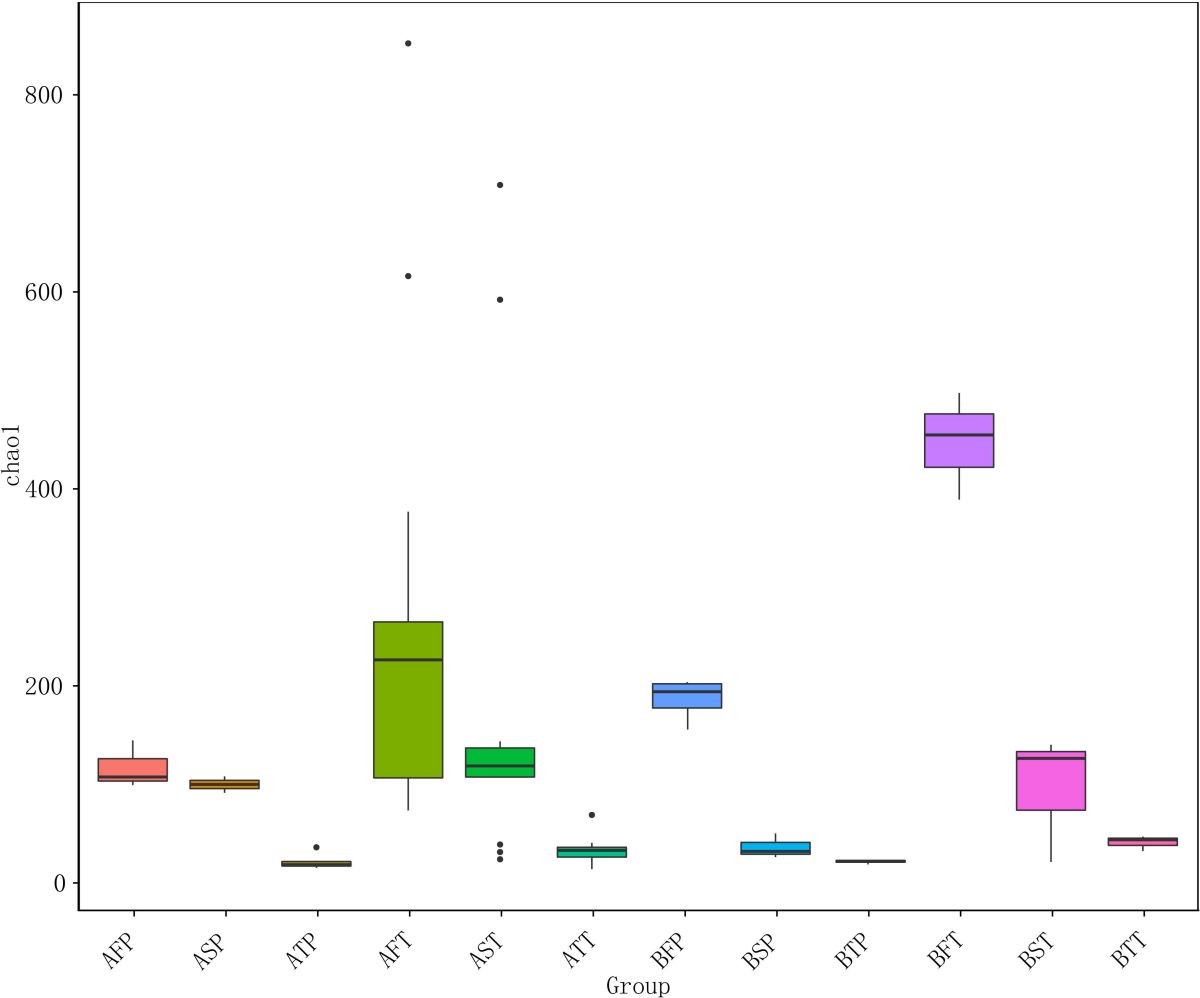
Chao 1 diversity index of different samples. For the first letter, A indicates IVF pregnancy, B indicates natural pregnancy; for the second letter, F indicates first trimester, S indicates second trimester, T indicates third trimester; for the third letter, P indicates preterm delivery, T indicates term delivery.

Among women who conceived *via* IVF (A) or naturally (B), the number of species and diversities in the bacterial community in those who delivered at term (T) were significantly higher than those in women who delivered before term (P) throughout pregnancy (F, first trimester; S, second trimester; T, third trimester), regardless of the pregnancy pattern. The numbers of the observed species were as follows: AFT > AFP, AST > ASP, ATT > ATP, BFT > BFP, BST > BSP, and BTT > BTP (**Figure 2**), indicating that preterm delivery can be predicted by the diversity of vaginal bacterial communities, regardless of whether the pregnancy is natural or *via* IVF. In addition, the bacterial communities could be more easily detected in early gestation than in the next two trimesters; the number of bacteria sharply declined during late gestation, and AFT > AST > ATT, AFP > ASP > ATP, BFT > BST > BTT, BFP > BSP > BTP (**Figure 2**).

Among women who conceived naturally as well as in those who conceived after IVF, and in those who delivered at term (T) and before term (P), the vaginal bacterial communities in the first trimester were higher than those in the other two trimesters. This indicated that the first trimester was a critical period during pregnancy, and the diversity of vaginal bacterial communities decreased with increasing gestational age in all samples (**Figure 2**).

The results indicated that conception after IVF could be associated with significantly decreased number of VMB in the first trimester, and that reduced microbial diversity in the vagina could be associated with an increased risk of preterm birth.

### 3.3 Vaginal bacterial diversity in different samples

**Figure 3:**
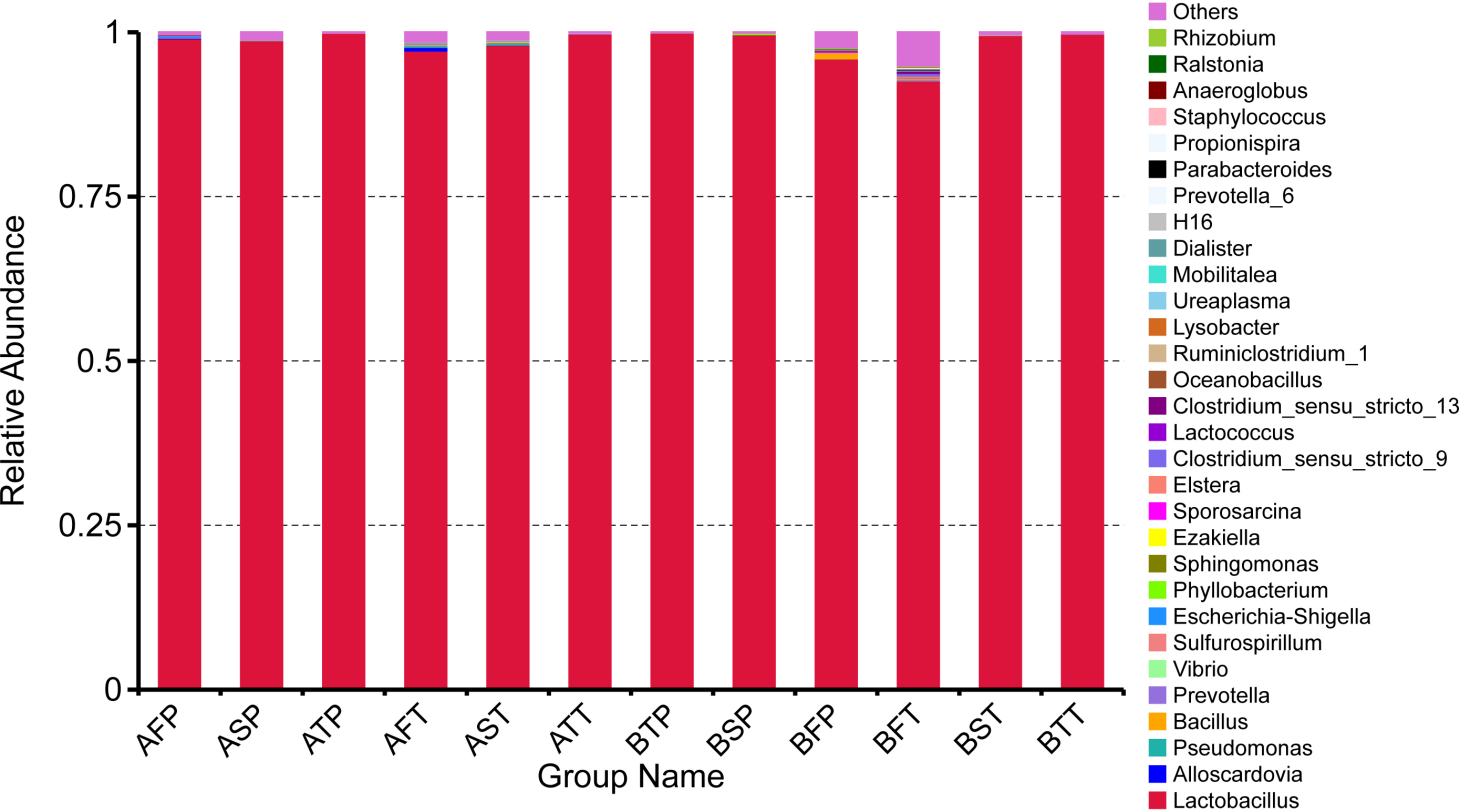
Relative abundance of top 30 genera in different samples. The distribution of the top 30 genera of all samples was analyzed.

At the genus level, *Lactobacillus* was dominant in all the samples (>93%), which was consistent with previous reports (34, 35). However, we found some differences among these samples (**Figure 3**) with regard to bacterial diversity. In the group of participants who conceived after IVF, the vaginal bacterial diversity in those who delivered before term was lower in the first and second trimesters, compared with participants who delivered at term; however, there were no differences in the bacterial diversity in the third trimester between the groups. In the group of participants who conceived naturally, the vaginal bacterial diversity in those who delivered before term was lower in the first trimester, compared with participants who delivered at term; however, there were no differences in vaginal bacterial diversity in the second and third trimesters between the groups.

In all women who conceived naturally, regardless of whether they delivered at term or before term, bacterial diversity was obvious in the first trimester. Thus, detection of vaginal bacteria might be helpful to monitor the risk of preterm birth in pregnant women. We found that among the top 30 genera, *Lactobacillus* was dominant in all samples, *Alloscardovia* was found only in participants who conceived after IVF, no *Bacillus* was found in the third trimester in all samples, *Vibrio* appeared only in AFT, *Sulfurospirillum* and *Propionispira* were detected only in BFT, *Sporosarcina* was detected only in AFT and AST, *Lactococcus* was found only in BFT and BFP, and *Oceanobacillus* was found only in BFP. In women who conceive after IVF, *Vibrio* and *Sporosarcina* might act as negative biomarkers for the risk of preterm delivery. As per our research, in women who conceive naturally, *Oceanobacillus* can act as a positive biomarker, and *Sulfurospirillum* and *Propionispira* can act as negative biomarkers for the risk of preterm delivery.

### 3.4 Bacterial diversity is important in naturally pregnant women who deliver at term

**Figure 4:**
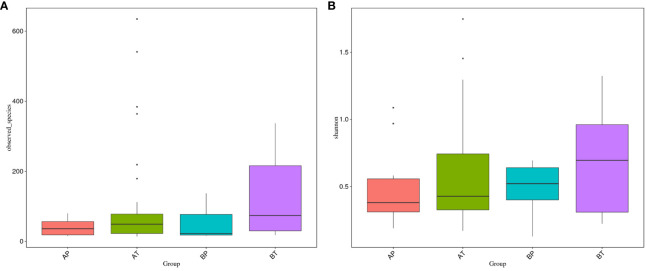
Vaginal bacteria diversities among different groups. **(A)** Women who were pregnant after IVF; **(B)** naturally pregnant women; P, pregnant women who delivered before term; T, pregnant women who delivered at term.

**Figure 5:**
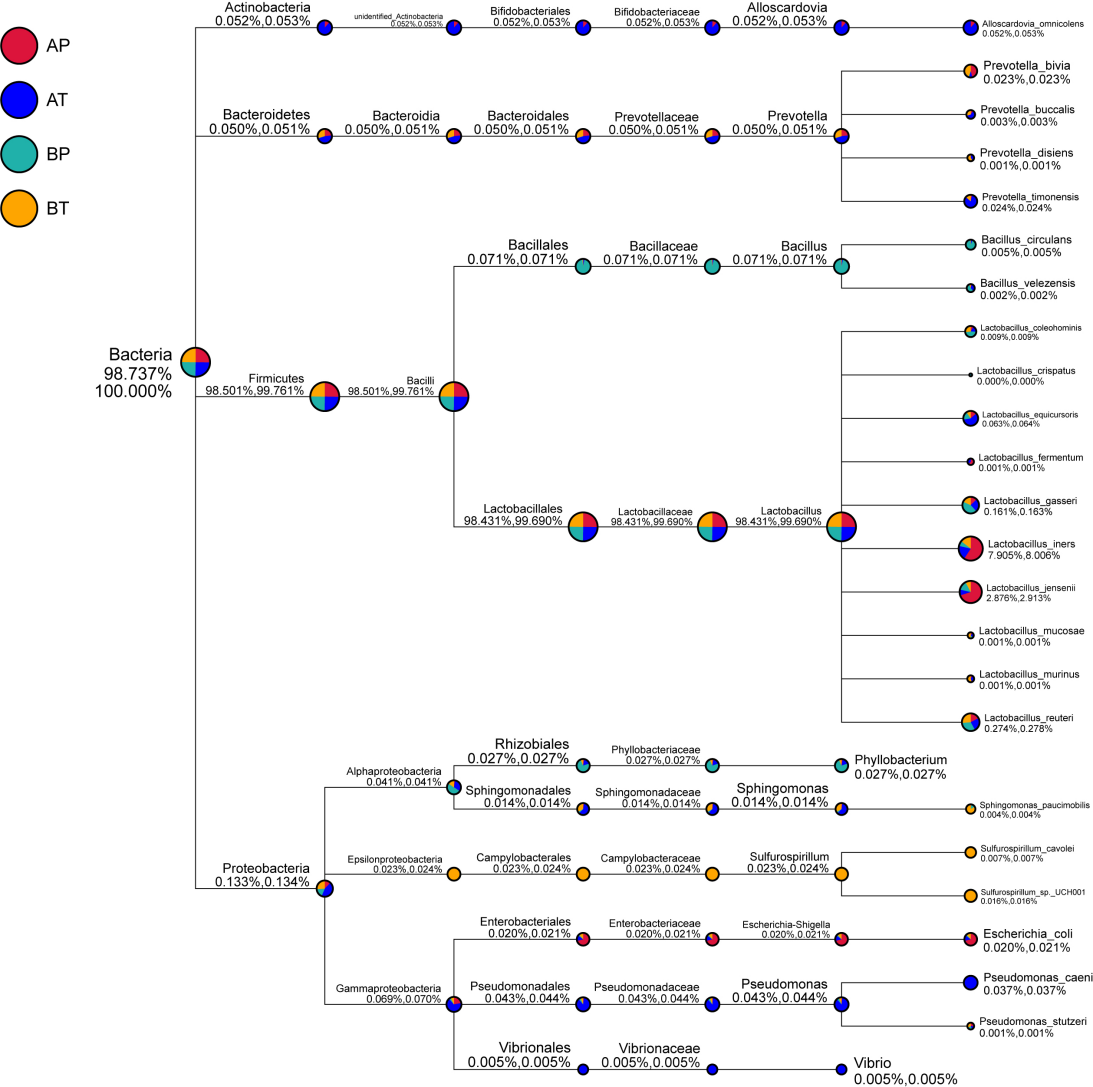
Taxonomic tree of specific species in each group. A, women who were pregnant after IVF; B, naturally pregnant women; P, pregnant women who delivered preterm; T, pregnant women who delivered at term.

We further analyzed the differences of vaginal bacterial diversities among the different groups: between participants who conceived after IVF (A) and those who conceived naturally (B), and between women who delivered before term (P) and those who delivered at term (T) (**Figure 4**). We observed that group BT had the highest number of observed species, and although groups BP and AT had a similar number of species, group AT had higher diversity than group BP did, and group AP had the lowest number of observed species and diversity among the four groups, indicating that bacterial diversity might play an important role in a healthy pregnancy. *Firmicutes* was dominant (98.501%) in all the samples, followed by *Proteobacteria* (0.133%), *Actinobacteria* (0.052%), and *Bacteroidetes* (0.050%). *Actinobacteria* appeared only in groups AT and AP (mostly in group AT), and *Sulfurospirillum* was found only in group BT. *Vibrio* was found only in group AT (**Figure 5**). The diversities and numbers of microorganisms were important for further outcome of pregnancy, especially for patients who conceived after IVF. Hence, standard protocols should be established to support a shift of vaginal microbiota during IVF therapy.

### 3.5 Differential species analysis in different groups

**Figure 6:**
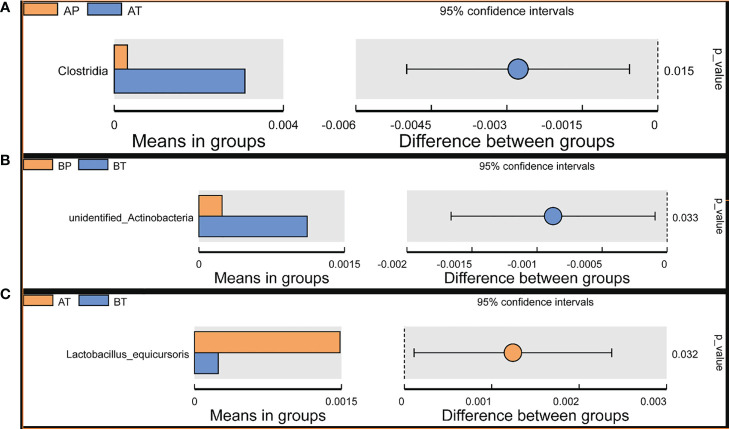
T-test analyses of different microbes between the groups. T-test analysis of different microbes between the groups. **(A)** AP and AT; **(B)** BP and BT; **(C)** AT and BT.

Student’s *t*-test was performed to determine the significant difference between the bacterial species in all the groups (*P* < 0.05), and the results showed significant differences between groups AP and AT. *Clostridia* were a significant differential species and were dominant in group AT; between groups BP and BT, unidentified *Actinobacteria* were a significant differential species and were dominant in group BT; between groups AT and BT, *Lactobacillus equicursoris* was a significant differential species and was dominant in group AT (**Figure 6**). Thus, *Clostridia* and *Lactobacillus equicursoris* can act as positive biomarkers for preterm birth in patients who conceive after IVF.

**Figure 7:**
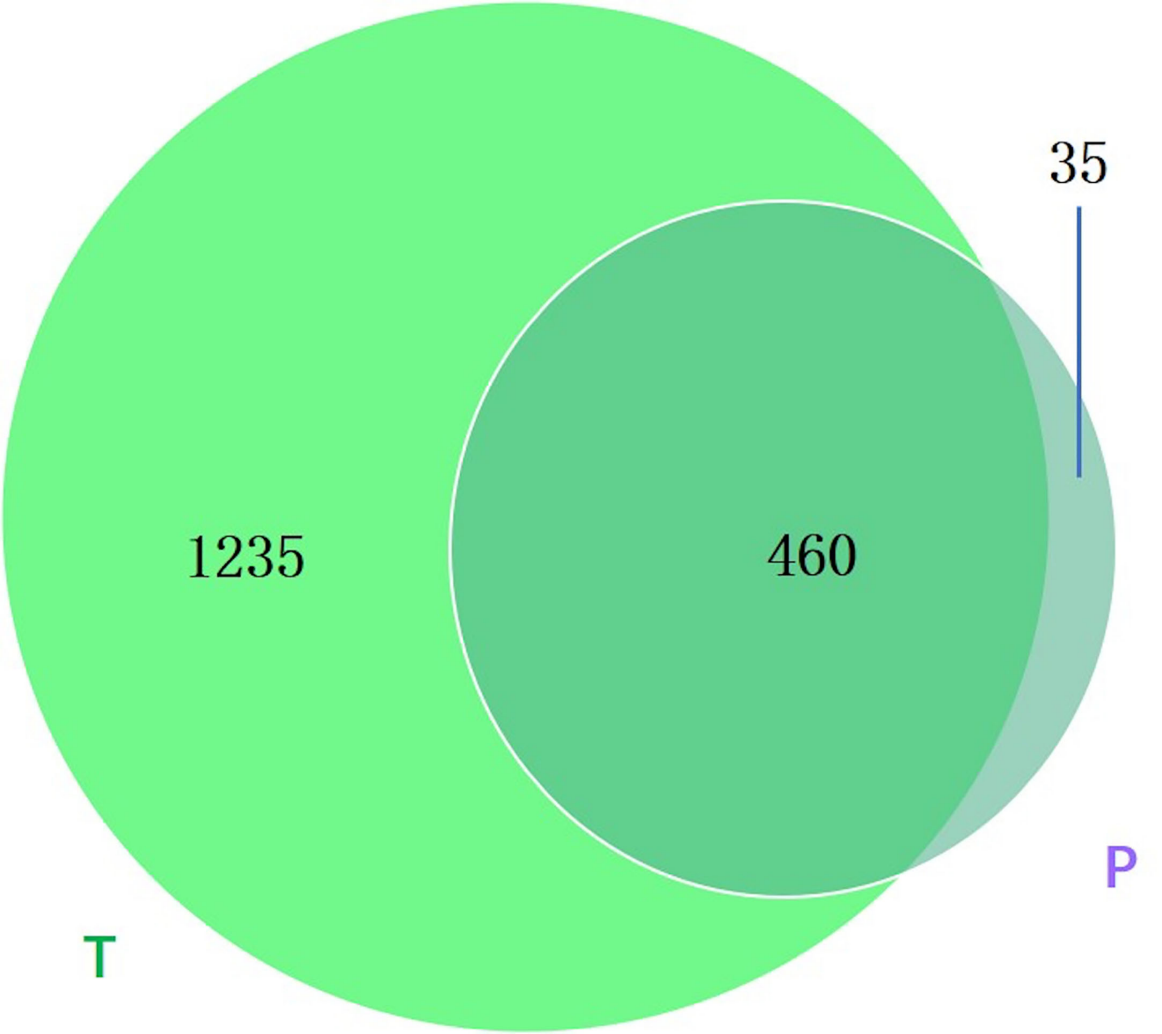
Venn diagram showing the number of microbes in different groups. The sharing numbers indicate the same microbes between the two groups, and the sole number represents microbes that appeared in only one group. P indicates preterm delivery and T indicates term delivery.

Between groups P and T, 35 bacterial species were found only in group P (**Figure 7**), and the annotation results indicated that they were novel species and may act as biomarkers for the risk of preterm birth.

## 4 Discussion

Preterm birth is a major contributor to infant death and neonatal mortality, which is a global concern, and researchers have mostly focused on finding an efficient approach to prevent preterm delivery (36, 37). Currently, IVF is an effective method for conception in women who fail to conceive naturally; however, IVF is associated with an increased risk of preterm birth. Dynamic changes in the VM of women who become pregnant through IVF are still poorly understood. As the importance of microorganisms for human health is well known (38–40), vaginal bacteria have been extensively studied in recent years, with an aim to determine their roles in pregnant women (13, 41–44). Previous researchers have mostly focused on the association between vaginal bacteria and preterm birth among American, African, and European women (21, 22, 45, 46), and little is known about the relationship between the vaginal microbiome and delivery pattern among women from Northern China who conceive *via* IVF.

In this study, using high-throughput sequencing technology (41, 47), we characterized the vaginal microbiome of women from Northern China who conceived *via* IVF and those who conceived naturally. We focused on the relationships between VMB and pregnancy after IVF, especially their effect on pregnancy outcome, to determine whether any taxa distinguished women who conceive after IVF from those who conceive naturally, and women who deliver before term from those who deliver at term.

We found that the community richness and diversity of VMB was lesser in women who conceived after IVF than in those who conceived naturally throughout pregnancy, regardless of the delivery pattern, suggesting that the VMB were more stable in naturally pregnant women than in those who were pregnant after IVF. The number of observed species was lesser in women who conceived *via* IVF and delivered before term than in women who delivered at term, suggesting that the vaginal bacterial communities associated with term birth were more stable than those associated with preterm birth. The diversity and richness of the vaginal bacterial communities diversity decreased in each trimester (first trimester > second trimester > third trimester), indicating that early pregnancy is a significant period for the vaginal bacterial community, and that the fluctuation of vaginal bacteria in the first trimester may be a biomarker for preterm birth.

A previous study reported that the genus *Lactobacillus* was predominant in the vagina of naturally pregnant women (42), and lower vaginal levels of *Lactobacillus crispatus* indicate a higher possibility of premature birth. Fettweis (48) reported Shannon Diversity is higher in women who deliver preterm. In that study, they used the data from women of African ancestry. Subsequently confirmed in other studies, the Vaginal microbiome profiles of women of African, European and Asian ancestry differ significantly. In Richard W. Hyman (43) study from Stanford University, they showed Chao I analysis is significantly distinguished by race/ethnicity. Black people are much higher than Hispanic, Caucasian and Asian. Chao I value of Caucasian and Asian are close and much lower than Black. And When they measure Shannon Diversity Index for Caucasian, they found Shannon Diversity is higher in woman who deliver at term which is consistent with us. We think microbiota diversity is influenced by many factors included ancestry and area.

Consistent with this observation, our data also showed that this genus was dominant in women who were pregnant after IVF. To determine the specific taxa in women who were pregnant after IVF, the details of vaginal bacteria were analyzed; we observed that *Alloscardovia* was found only in women who were pregnant after IVF, *Vibrio* was found only in AFT, and *Sporosarcina* was detected only in AFT and AST. Multiple group analysis showed that the number of observed species and diversity were in the order BT > AT > BP > AP, indicating that VMB play important roles in a healthy pregnancy. *Actinobacteria* were detected only in groups AT and AP (especially in group AT), *Vibrio* was found only in group AT, and *Sulfurospirillum* was found only in group BT. *Alloscardovia* was specifically detected in women who conceived *via* IVF and could act as a positive biomarker, whereas *Vibrio* and *Sporosarcina* could act as negative biomarkers for the risk of preterm birth. *Oceanobacillus* might act as a positive biomarker, whereas *Sulfurospirillum* and *Propionispira* might act as negative biomarkers for the risk of preterm birth in women who conceive naturally.

Oceanobacillus, a member of the Baciliaceae, was isolated from deep-sea sediments by Lu et al. (49) using O. iheyensis as a model species and was gram positive. Studies have shown that colonizing vaginas in pregnant mice causes vaginal inflammation and may alter cervix functions and integrity (50). Previous work has validated the breakdown of the cervical epithelial barrier due to inflammatory damage (51).

Kumar M and Hočevar K (52, 53) identified that increased bacterial diversity/BV was positively associated with PTB.in Hočevar K’ study of shown that the predominant bacterial families were Lactobacillaceae (77.9%), in our finding that Lactobacillaceae dominant in all the samples(>93%), that mean there are great differences in the results of vaginal microecology between the two populations. And the data of Kumar’reaserch are more broadly applicable to Asian women in ethnically-diverse populations in high income nations.

The main reason for the different research results may be that all our samples were after IVF and some vaginal operations were performed before pregnancy.

So far the characteristics of vaginal Microecology in pregnant women after IVF have not been described.

19 women had conceived after IVF after a long protocol of conventional solutions of fresh-cycle IVF, our sample pregnant women are completely infertile due to male factors, avoiding the changes of vaginal microecology caused by changes in drug and hormone levels. However, before becoming pregnancy, these pregnant women will have repeated vaginal ultrasonography, vaginal flushing and transvaginal fornix oocyte collection, which will destroy the integrity of vaginal flora, These reasons may be the reasons for the differences in vaginal flora samples. Because our samples are all male factors, this also avoids the impact of changes in maternal hormone levels on vaginal flora.

Before pregnancy, vaginal flora structure was destroyed after vaginal flushing. So after pregnancy, vaginal flora structure also means reestablishment. For this reason perhaps the number of species and diversities in the bacterial community in those who delivered at before term throughout pregnancy were lower.

These results are currently observed in our research. The specific mechanism is not very clear. We will study it in the next research.Second, due to the small number of pregnant women with simple male factor and insufficient sample size, the results may also be different. We will increase sample size in the next research.

We recruited 19 volunteers in IVF group and 6 volunteers in Natural conception group and collected vaginal samples from each volunteer in three periods. These samples are very precious and can effectively remind us of the relationship between vaginal bacteria and pregnancy outcomes. In our results, we found vaginal samples collected during first trimester showed richer differences and more predictive value for pregnancy outcomes. In addition, these data indicates that Alloscardovia, Vobrio and Sporosarcina have great potential in predicting pregnancy outcomes who pregnant by vitro fertilization. While Oceanobacillus, Sulfurospirillum and Propionispira have great potential in predicting the risk of preterm birth in women who conceive naturally.On the other hand, the sample size in our research is not enough to conclude these bacteria strains we suggested could be biomarker for preterm birth. And the mechanism of these bacteria strains on preterm birth is unclear. But our data is a powerful indicator.Although the exact causal relationship remains to be determined, our results confirm an association between some bacteria and preterm birth. However, the richness, diversity, and stability of the microbiome may be important during pregnancy. Our finding is consistent with previous studies (54, 55).

In summary, our study showed that compared with naturally pregnant women, women who conceive *via* IVF and those who deliver before term show lesser richness, diversity, and stability of VMB. Therefore, standard protocols should be established and used to support a shift of VMB.

## Data availability statement

The datasets presented in this study can be found in online repositories. The names of the repository/repositories and accession number(s) can be found below: https://www.ncbi.nlm.nih.gov/, PRJNA728871.

## Ethics statement

The studies involving human participants were reviewed and approved by Dalian Women’s and Children’s Medical Center (approval number 20160021). The patients/participants provided their written informed consent to participate in this study.

## Author contributions

YT: responsible for the experimental concept, data collection, drafting papers; QS: responsible for the data collection, drafting papers; XS: Responsible for the important revision of the thesis; ZW: Responsible for the important revision of the thesis and for the important revision of the thesis. All authors contributed to the article and approved the submitted version.
